# Use of the DELTA Model to Understand the Food System and Global Nutrition

**DOI:** 10.1093/jn/nxab199

**Published:** 2021-06-30

**Authors:** Nick W Smith, Andrew J Fletcher, Lakshmi A Dave, Jeremy P Hill, Warren C McNabb

**Affiliations:** Riddet Institute, Massey University, Palmerston North, New Zealand; Sustainable Nutrition Initiative, Riddet Institute, Massey University, Palmerston North, New Zealand; Riddet Institute, Massey University, Palmerston North, New Zealand; Sustainable Nutrition Initiative, Riddet Institute, Massey University, Palmerston North, New Zealand; Fonterra Research and Development Centre, Palmerston North, New Zealand; Riddet Institute, Massey University, Palmerston North, New Zealand; Sustainable Nutrition Initiative, Riddet Institute, Massey University, Palmerston North, New Zealand; Riddet Institute, Massey University, Palmerston North, New Zealand; Sustainable Nutrition Initiative, Riddet Institute, Massey University, Palmerston North, New Zealand; Fonterra Research and Development Centre, Palmerston North, New Zealand; Riddet Institute, Massey University, Palmerston North, New Zealand; Sustainable Nutrition Initiative, Riddet Institute, Massey University, Palmerston North, New Zealand

**Keywords:** systems modeling, micronutrients, sustainability, mass balance, nutrient adequacy, mathematical modeling

## Abstract

**Background:**

Increasing attention is being directed at the environmental, social, and economic sustainability of the global food system. However, a key aspect of a sustainable food system should be its ability to deliver nutrition to the global population. Quantifying nutrient adequacy with current tools is challenging.

**Objective:**

To produce a computational model illustrating the nutrient adequacy of current and proposed global food systems.

**Methods:**

The DELTA Model was constructed using global food commodity balance sheet data, alongside demographic and nutrient requirement data from UN and European Food Safety Authority sources. It also includes nutrient bioavailability considerations for protein, the indispensable amino acids, iron, and zinc, sourced from scientific literature.

**Results:**

The DELTA Model calculates global per capita nutrient availability under conditions of equal distribution and identifies areas of nutrient deficiency for various food system scenarios. Modeling the 2018 global food system showed that it supplied insufficient calcium (64% of demographically weighted target intake) and vitamin E (69%), despite supplying sufficient macronutrients. Several future scenarios were modeled, including variations in waste; scaling up current food production for the 2030 global population; plant-based food production systems; and removing sugar crops from the global food system. Each of these scenarios fell short of meeting requirements for multiple nutrients. These results emphasize the need for a balanced approach in the design of future food systems.

**Conclusions:**

Nutrient adequacy must be at the forefront of the sustainable food system debate. The DELTA Model was designed for both experts and nonexperts to inform this debate as to what may be possible, practical, and optimal for our food system. The model results strongly suggest that both plant and animal foods are necessary to achieve global nutrition. The model is freely available for public use so that anyone can explore current and simulated global food systems.

## Introduction

The global food system is complex, involving numerous inputs, outputs, and feedback loops (**Supplemental Figure 1**). Understanding it and identifying opportunities for improvement requires a comprehensive view of the whole system, which is not easily achieved. However, increasing calls for greater environmental sustainability and nutrition security in the global food system have motivated attempts to rigorously analyze and model subsets of its dynamics (1–7).

The sustainability of the global food system encompasses multiple aspects. The following definition was drawn from the UN High-Level Task Force on Global Food and Nutrition Security (8):

A sustainable food system is a food system that delivers food and nutrition security for all in such a way that the economic, social, and environmental bases to generate food security and nutrition for future generations are not compromised.

Thus, among other essential factors, a sustainable global food system must ensure nutrient adequacy for the global population; that is, that adequate levels of all essential nutrients are provided within upper and lower bounds to prevent deficiencies and avoid toxicity (9). The increasing numbers of hungry or undernourished individuals globally [690 million people hungry in 2020, around 60 million higher than 2015 (10); 820 million facing some form of undernourishment (11)] demonstrates that the current system is not providing the required amounts of each essential nutrient to all individuals, either due to individual choice or societal forces (4, 6). Although part of this challenge is the unequal distribution of food, it is not fully understood whether the current food system produces sufficient nutrients to nourish the global population even under conditions of equitable distribution (12–14). Without this sufficiency, global sustainable nutrition cannot be achieved.

It is important that nutrient adequacy is assessed for all nutrients essential to human health. Although high-level discussion of healthy sustainable diets in the literature implies nutrient adequacy (2–7), the large number of nutrients essential for health make inclusion of all these nutrients in food system models challenging (1). As a result, some studies consider energy or protein only (15–18), some utilize calculated nutrient metrics (19–23), whereas others include a large number of individual nutrients (24–27). Part of the difficulty in assessing nutrient adequacy is in obtaining data for the nutrient content of foods and intake requirements. The European Food Safety Authority (EFSA) has produced population reference intake values for 11 macronutrients and micronutrients, as well as average intakes associated with good health for a further 21 (28). However, even this level of detail does not include recommendations for indispensable amino acid (IAA) intakes [(29)], considering only total protein. A full analysis of nutrient adequacy must take all essential nutrients into account (30).

To understand the nutritional sustainability of the global food system, we have developed the DELTA Model, which takes global food system scenarios as its inputs and calculates the nutrients available to the global population. The nutrients available are scaled according to the bioavailability of nutrients from different food sources, where data is available to facilitate this. Finally, the available nutrients are compared with a demographically weighted, globally averaged target daily intake for each nutrient assuming equal distribution of food, allowing the nutrient adequacy of the food system to be determined.

Using this framework, the DELTA Model allows for comparison between different future global food system scenarios in terms of nutrition, to be used in combination with investigations into the other aspects of sustainability. The DELTA Model is so named as it enables side by side comparisons of the mathematical difference (or **Δ**) between modeled scenarios, including those changes required to move from insufficient nutrient supply to a food system that can deliver global nutrient adequacy.

## Methods

### Overview

**FIGURE 1:**
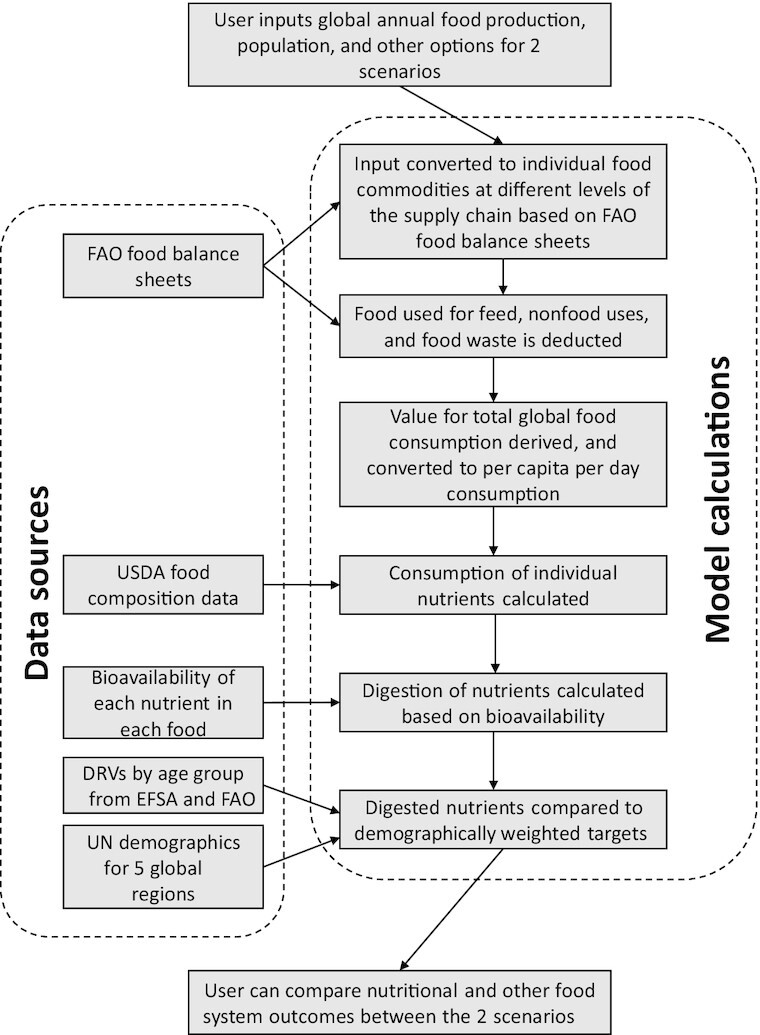
Flow diagram of the general structure of the DELTA Model. Full details of the model calculations can be found in Supplemental files 1–3. DRV: dietary reference value; EFSA: European Food Safety Authority.

The DELTA Model was constructed in R (version 4.0.2), from a combination of publicly available databases and in-house modeling. The general structure of the model is displayed in **Figure 1**; a detailed description of the model calculations can be found in **Supplemental files 2** and **3**. The DELTA Model and further supporting material can be accessed online (31).

The DELTA Model was designed to allow visual comparison of the nutritional performance of global food system scenarios. The user defines a scenario by entering values for several variables, including primary production of 121 food commodities, a scenario year that defines the global population, and food waste (in the supply chain and in-home). For accessibility, the 121 food commodities are aggregated into 15 major food groups, 6 animal-sourced (ruminant meats, poultry meats, other meats, eggs, dairy, and fish/seafood) and 9 plant-sourced (cereals, fruit, nuts, oilcrops, pulses, starchy roots, sugar, vegetables, and other plants). The nutritional performance of this scenario can then be compared with alternatives.

### Nutrition calculations

The DELTA Model takes the user inputs for global annual food production and distributes these into individual food items according to their distribution in the 1998–2017 FAO food balance sheets (FBS) (32). The 2018 annual food production was derived from a weighted linear interpolation of previous years’ production, as described in Supplemental file 3. For example, production of the food group “Fruits” is distributed proportionally into individual food items (e.g. apples, bananas) based on their proportional distribution in the FBS.

The model next deducts feed uses from those food items that are used for animal feed, as well as supply chain waste and nonfood use of food items, in accordance with the proportion found in the FBS.

This yields a value for the total amount of each food item available to consumers per year. From this is deducted the inedible portion of relevant food items [e.g. vegetable skins/peels, animal bones, according to refs (14, 33–35)], and the proportion that is wasted in-home. The in-home waste proportion is specific to food groups and derived from regional estimates by the FAO (36). This yields a value for the total edible amount of each food item available to the global population each year, which is rescaled to provide a per capita daily allocation of the food item. This per capita daily allocation assumes equal distribution of food to all individuals; the DELTA Model does not consider the variations in food distribution present in the world. The rationale for this is that if global nutrient adequacy cannot be achieved in a scenario under the assumption of equal food distribution, then it cannot be achieved in the presence of unequal distribution.

The composition of each modeled food item was taken from the USDA database (37). Combining these with the daily food item allocation value gives a per capita daily allocation for each nutrient. However, not all ingested nutrients are absorbed, and the extent of nutrient absorption is dependent on the food source (29). Data on nutrient absorption and bioavailability is not available for all nutrients in all foods, but digestibility data is available for protein and the IAA for a range of foods (38, 39). Therefore, the total protein and amino acid content of a food is multiplied by a bioavailability coefficient (between 0 and 1) according to the food item from which the nutrient was derived, reflecting the proportion of the nutrient that is digestible. The use of digestibility data does not capture all aspects of bioavailability (e.g. the ratio of amino acids in a food), but allows the model to consider the differences in protein quality between foods.

For iron and zinc, insufficient data was available for the bioavailability or digestibility of these nutrients in foods to allow the same structure to be applied. Instead, the DELTA Model contains higher target intakes for these nutrients, sourced from WHO recommendations (40), to be used in scenarios where little or no animal-sourced foods are produced. Details on the derivation of the bioavailability coefficients can be found in **Supplemental file 1**.

In order to compare the nutrition values of the modeled scenario to dietary reference value (DRV) data, DRVs for 29 nutrients were obtained from the EFSA and the FAO (28, 41). These 29 included the dietary macronutrients, 7 IAA, and 17 vitamins and minerals. The full list can be seen in Supplemental file 1 or the online version of the model.

Demographic information for 21 age groups of both genders in 5 global regions was obtained from the FAO databases for past and future forecast populations (42). The DRV and demographic information were combined to give a demographically weighted target daily intake for the average global citizen, as well as upper and lower limits for safe intakes where data was available. The values of this target are then compared to the modeled scenario nutrient profile, and this comparison is returned to the user.

## Results

The DELTA Model was designed to be used by both experts and nonexperts to explore current and future scenarios for the global food system. The role of the DELTA Model is to identify areas of the global food system in need of improvement, to inform the sustainable nutrition debate, and as an educational tool for users. It is not an optimization tool to identify prescriptive changes that should be implemented in the future. Instead, a number of example uses and results are presented here to illustrate the capacity of the DELTA Model.

### Examining the current global food system

The baseline data set for the DELTA Model is the 2018 global population and food production, extrapolated from the 1998–2017 FBS (see **Supplemental file 4** for derivation and model inputs in each scenario). From this data set, it was possible to examine how the 2018 food system would have met the nutrient requirements of the population under conditions of equal food availability. **Table 1** shows an overview of the results of the DELTA Model for 2018, compared with a second scenario in which the 2018 food system was applied to the forecast 2030 global population of 8.6 billion.

**TABLE 1:** DELTA Model results using the 2018 food system to feed the 2018 population or the forecast 2030 population

	2018 population (7.6 billion)	2030 population (8.6 billion)
Total biomass production leaving farms/fisheries, billions of tons/y	10.58	10.58
Total food supply after waste, billions of tons/y	4.64	4.6
Amount of total biomass above used as animal feed, billions of tons/y	1.5	1.5
Energy supply (energy target), kcal/person⋅ d	2502 (2160)	2244 (2166)
Protein supply (protein target), g/person⋅ d	61 (45.4)	54.7 (46.1)
Fat supply (fat target), g/person⋅ d	76.1 (60.1)	68.1 (60.3)
Nutrient gaps >5%, % of target daily intake		
Calcium	36	43
Vitamin E	31	42
Iron	—	11
Potassium	—	12
Riboflavin	—	6
Vitamin A	—	9
Vitamin B-12	—	6

Total biomass production and feed use were identical between the two scenarios, as it was assumed that only the global population had changed. The total food supply after waste was lower for the 2030 scenario; this was due to increased nonfood uses (e.g. biofuel production) scaled to the larger population.

The main difference between the two scenarios was in the nutrient gaps. The nutrient gaps show the deficiency between the per capita global availability of a nutrient and the target daily intake value. Only gaps greater than 5% of the target value are displayed; smaller gaps were not considered to be material from the degree of accuracy of the model. In the 2018 scenario, global food production delivered insufficient calcium and vitamin E. Thus, even with the equal distribution of food assumed by the model, the 2018 global food system would have left its population deficient in these nutrients.

Clearly, these nutrient gaps would grow if the same amount of food were distributed over a larger population, as shown in the 2030 scenario. The nutrient gaps for calcium and vitamin E grew, while iron, potassium, riboflavin, vitamin A, and vitamin B-12 were added to the list of deficient nutrients.

**FIGURE 2:**
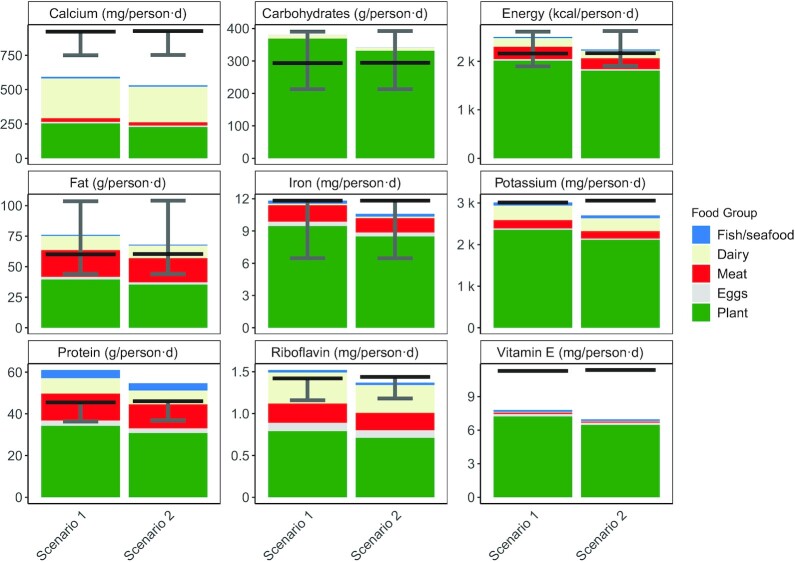
Comparison between the nutrients supplied in 2018 baseline scenario (Scenario 1 in the figure) and the same food production system applied to the 2030 population (Scenario 2). A selection of essential nutrients is shown. The bars show per capita per day nutrient supply in the specified unit, with the black horizontal lines showing the demographically weighted global target daily intake value for each nutrient. Where available, demographically weighted upper and lower safe intake values for each nutrient are shown by the gray error bars.

**Figure 2** illustrates the nutrient differences between the two scenarios. Data for calcium requirements are more detailed than for vitamin E, hence the display of a lower safe intake value and a target value for calcium, whereas only the latter is present for vitamin E (28). The importance of a lower safe intake value can be seen when comparing calcium and iron in the 2030 scenario: although neither reached the target daily intake value, iron availability was above the lower safe intake value, and thus less concerning than the calcium deficiency.

The globally averaged target daily intake values differed between the two scenarios (Figure 2). This was due to changes in population structure, with a higher ratio of adults to children and of women to men in the 2030 forecast population. Note that, in both scenarios, energy, protein, and fat availability were all greater than the target values for these macronutrients. This emphasizes the need to assess nutrient adequacy on a micronutrient level, as deficiencies may exist despite macronutrient surpluses.

### Food waste

The reduction of food waste forms part of the UN Sustainable Development Goals [Target 12.3; (43)]. The DELTA Model does not consider food losses before leaving the farm or fishery (e.g. crops not entirely harvested), but does consider supply chain losses and in-home waste. To investigate changes in waste and losses with the DELTA Model, these quantities were scaled from the 2018 levels to examine the effect on nutrient deficiencies. **Table 2** shows the results of these simulations using the 2018 baseline data set.

**TABLE 2:** Impact of variations in food waste in-home and food losses along the supply chain on nutrient gaps using the 2018 baseline data set. Nutrient gaps of <5% are not shown. All values specified are the magnitude of the nutrient gap as a percentage of target daily intake

	0 × supply chain loss	0.5 × supply chain loss	1 × supply chain loss	1.5 × supply chain loss
0 × in-home waste				
Calcium	24	26	28	30
Vitamin E	20	23	25	27
0.5 × in-home waste				
Calcium	28	30	32	34
Vitamin E	23	26	28	30
1 × in-home waste				
Calcium	32	34	36	37
Potassium	—	—	—	5
Vitamin E	27	29	31	33
1.5 × in-home waste				
Calcium	36	38	40	41
Fiber	—	—	—	5
Iron	—	5	9	12
Potassium	—	—	—	13
Vitamin A	—	—	—	8
Vitamin E	30	32	34	36
Zinc	—	—	7	10

Regardless of waste and loss reduction, there were no instances in which the 2018 food system could deliver nutrient adequacy for the global population. At zero waste and losses, the nutrient gaps for calcium and vitamin E were ≥20%. Increasing waste and losses led to increasing degrees of nutrient deficiency. In the 2018 data set, milk and oil crop foods were the major sources of calcium and vitamin E, respectively. Depending on the global region, the DELTA Model assumes that 0.1–15% of milk is wasted in-home, and 1–4% of oil crop foods (36).

**FIGURE 3:**
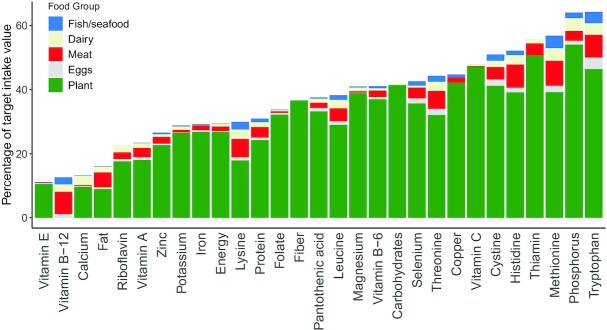
Comparison between levels of waste for each nutrient considered by the DELTA Model for the 2018 baseline scenario. The bars show total nutrient waste and loss as a percentage of target daily intake.

The DELTA Model also allows users to investigate the nutrients wasted. In the 2018 baseline scenario, for all nutrients except vitamin B-12, plant foods were responsible for most nutrient waste and losses (**Figure 3**). For 5 nutrients, the total wasted or lost was >50% of the target daily intake value (phosphorous, thiamin, cystine, methionine, and tryptophan), meaning that any deficiencies in these nutrients could be effectively tackled by reducing food waste and loss. Conversely, waste and losses of vitamin B-12, vitamin E, and calcium were low (11–13% of the target values), thus deficiencies in these nutrients would be better remedied with greater availability of food, rather than reduced food waste.

### 2030 scenarios

The 2030 population is forecast to be ∼12% higher than the population in the 2018 baseline data set, with a higher average age (42). As seen earlier in this section, retention of the food production levels of the 2018 food system resulted in increasing nutrient gaps as the population increased. **Table 3** shows several examples in which the DELTA Model was used to analyze the nutritional outcomes of various future scenarios.

**TABLE 3:** DELTA Model results for various simulated future scenarios with the forecast global populations of 8.6 billion in 2030 and 9.7 billion in 2050

	Baseline data set (2018)	Scale-up (2030)	No meat (2030)	No sugar (2030)	Half waste (2050)
Total biomass production leaving farms/fisheries, billions of tons/y	10.58	11.85	12.04	11.85	10.58
Total food supply after waste, billions of tons/y	4.64	5.21	6.21	6.84	5.12
Amount of total biomass above used as animal feed, billions of tons/y	1.5	1.68	0.47	1.68	1.5
Nutrient gaps >5%, % of target daily intake					
Calcium	36	36	32	26	46
Iron	—	—	26	—	12
Potassium	—	—	—	—	15
Riboflavin	—	—	—	—	11
Vitamin A	—	—	—	—	12
Vitamin B-12	—	—	35	—	14
Vitamin E	31	33	24	7	48
Zinc	—	—	27	—	13
Nutrient excesses, % above safe upper limit value					
Carbohydrates	—	—	26	21	—
Energy	—	—	10	20	—

In the scale-up scenario, the 2018 food production was increased by 12% across all food groups, to match the population increase by 2030. Intuitively, nutrient gaps were similar to those seen in the 2018 data set.

The no meat scenario set all meat and seafood production to zero, whereas all remaining food groups were increased by 20% to achieve a similar total biomass production to the scale-up scenario. Total food supply increased at the expense of total feed supply, due to reduced demand for animal feed. The calcium and vitamin E gaps were reduced compared with the 2030 scale-up scenario, due to increased dairy and oil crop production and decreased use of these food groups as feed. However, nutrient gaps emerged for iron, vitamin B-12, and zinc. The excesses in energy and carbohydrates above the recommended upper limit indicate that obtaining the other nutrients in this scenario may only be possible with a high consumption of these macronutrients.

The next scenario investigated the removal of sugar from the global food system. In the baseline data set, sugar represents 22% of total postharvest biomass production (2.3 billion tons). Sugar products fill the top 3 places for carbohydrate density in this data set and make a negligible contribution to intake of other nutrients. Due to the low positive impact of sugar products on global nutrition, the scale-up scenario was modified by removing sugar and allocating the 2.3 billion tons of biomass proportionally across the remaining plant groups.

The no sugar scenario resulted in almost identical total biomass and feed compared with the scale-up scenario. However, the total food supply was increased by 1.63 billion tons, due to the lower mass yield of available food from sugar crops after processing and other uses (<10%) compared with other plant food groups. Moreover, the nutrient gaps in this scenario were reduced compared with the scale-up scenario. However, due to increased availability of plant foods, there were excesses for carbohydrates and energy.

The half waste scenario utilizes 2018 food production, with food waste halved for both supply chain and in-home losses. It applies this global food system to the 2050 population. The purpose of this scenario is to demonstrate that the current global food system can provide sufficient macronutrients to nourish a 2050 population; energy, protein, and fat targets are all met in this scenario, as are those for the IAA. However, gaps exist for several micronutrients. Reducing supply chain and in-home losses to zero in this scenario (not shown in Table 3), only resolved the iron, riboflavin, vitamin A, and zinc nutrient gaps. These results are partly due to the increase in population, but also due to the low waste of many micronutrients, as shown in Figure 3.

### The importance of considering nutrient bioavailability

The DELTA Model takes into consideration the differences in protein and IAA digestibility from different foods. The importance of this can be seen when simulating the ability of the no meat scenario above in nourishing the 2050 forecast global population.

Taking the simpler “composition only” view of nutrition, the no meat scenario met the 2050 global target values for IAA, with a 15% lysine surplus. However, when the digestibility of these amino acids in the available foods was considered, the IAA lysine was only supplied at the target intake concentration. A deficiency in this IAA would result in a diminished capacity for protein synthesis. Thus, considering food composition alone can lead to incorrect conclusions on the nutrient adequacy of a scenario, and thus on the changes to the food system required to fill nutrient gaps.

## Discussion

Increasingly, both the scientific and nonscientific media feature analyses of the global food system, individual diets, and recommendations on ways to improve these. These scientific publications generally take an environmental (16, 18, 44–48), economic (49, 50), or health perspective (51, 52), or some combination of these (2, 3, 15, 24, 53–55). Although each of these perspectives is necessary in the discussion of a sustainable food system, the most basic requirement of providing adequate nutrition for an increasing global population is not always discussed. We argue that nutrition should be a priority of the food system; an essential aspect of its sustainability. Further, nutrient adequacy should be assessed for all essential nutrients, not simply for macronutrients. The results of the DELTA Model demonstrate that micronutrient sufficiency can be challenged even in the presence of macronutrient surpluses, a phenomenon that has been observed in many parts of the developing world since the Green Revolution of the mid 20th century (56). Full discussion of nutrient adequacy is urgently required, given the current global challenges of malnutrition and those expected in the future (6, 10, 11).

Exceeding nutrient intake targets and nutrient adequacy are not identical concepts. Nutrient adequacy includes meeting nutrient targets without incurring the negative effects of overconsumption. A key example of this difference is in the oversupply of energy. It is possible to exceed all nutrient targets by consuming larger quantities of foods, but this can require excess energy intake. Thirteen percent of the global adult population was obese in 2016, with a 2.6% annual increase (10). The negative health consequences of obesity illustrate that overnutrition must be considered as much as undernutrition. Nutrient adequacy, including upper and lower bounds, should be a priority consideration of future food system discussion (27). The inclusion of upper and lower limits in the DELTA Model allows the user a more complete view of nutrition than single target values.

The results of the DELTA Model detailed above represent example uses of the model to investigate commonly discussed future possibilities for the global food system. Any number of simulated scenarios can be analyzed using the model to explore their possibility and practicality from a nutritional perspective. The scenarios are not presented as recommended courses of action, but rather as quantitative predictions to support or oppose hypotheses in the food system debate. Clearly, total removal of waste from the food system (Table 2) is not feasible, and reallocating sugar crop biomass to other plant food groups (Table 3) does not consider the land use implications of such a change. However, these scenarios provide a starting point for discussion: is halving food waste an appropriate goal from a nutritional perspective, or does a more achievable 25% reduction have a similar nutritional outcome? Is the current volume of sugar crop production optimal, given its minimal contribution to nutrition?

Models make simplifying assumptions, making them imperfect representations of a system. However, when considering highly complex systems, such as the global food system, models usefully combine the relevant aspects in a comprehensive and holistic way. A strength of the DELTA Model is its accessibility and flexibility, so that any number of scenarios may be investigated and areas requiring further research identified.

The 2018 baseline scenario provides important information for the sustainable nutrition debate. Firstly, sufficient macronutrients were produced in 2018 to nourish the global population under the assumption of equal nutrient distribution, as has been found for years previous to 2018 (12–14). Only calcium and vitamin E were not available in sufficient quantities. The DELTA Model calculates that sufficient iron, protein, and energy were produced globally, and yet iron-deficiency anemia was responsible for 54,200 deaths in 2015, and protein-energy malnutrition for 323,200 (57). This means that the global challenge of nutrient inadequacy is less a problem of insufficient production, and more an issue of distribution, access to food, and excess consumption (12–14).

It is worth noting that the DELTA Model deals with nutrient requirements, not demand. It is often repeated that demand for food will increase by as much as 70% by 2050 (58). Although that may be true, the DELTA Model examines nutrient requirements and shows that our current production system would adequately meet the energy and macronutrient needs of this future population. This highlights the difference between demand and requirement.

Perhaps the most important result presented above is the large gap between the amounts of calcium and vitamin E available from food and that which is required by the global population. Other nutrient gaps emerge in certain scenarios but are smaller and more easily remedied through increased food production and decreased waste. The large calcium and vitamin E gaps are coupled with low waste of these nutrients, a relationship that has been observed previously (59). In the DELTA Model, the food items with the greatest calcium to mass ratios are minor contributors to available food: seeds and spices. Milk has the greatest contribution to calcium in the 2018 baseline scenario but the model predicts that dairy production would need to at least double to remove the current calcium gap.

Likewise, for vitamin E, the 9 densest sources of this nutrient in the model are vegetable oils and oil crops. Although the model predicts a production increase of 60% would resolve this nutrient gap, the concomitant increase in caloric intake from higher oil consumption results in energy intakes above the safe upper limit. The current importance of milk and oil crops in the provision of calcium, vitamin E, and many other nutrients should motivate efforts to improve the efficiency of their production. However, the difficulties in resolving the calcium and vitamin E gaps through increased food production alone point to a need for alternatives, such as supplementary nutrition, to ensure that global nutrient availability meets global requirements.

The reliability of the DRVs varies between nutrients. For example, sufficient data exists to define an average requirement (that which meets the needs of half of the population), a population reference intake (likely to meet the needs of almost all healthy people), and an upper limit (above which adverse health effects are a risk) for calcium (28). Contrastingly, due to more limited data for vitamin E, only an adequate intake (based on population dietary observations and assumed adequate) and upper limit are available. Moreover, insufficient data exists to include the bioavailability of these nutrients in the model. This is a further outcome of the DELTA Model: the prediction that vitamin E is often deficient in the model scenarios, coupled with the limited data on what the bodily requirement and bioavailability of this nutrient are, highlights the need for further research on this nutrient.

The utility of the DELTA Model will grow over time through extension and development; the current version has several limitations. Foremost among these is omission of the other aspects of sustainability: environmental, economic, and social. Modeling studies are best suited to the environmental and economic aspects, and numerous models exist for these dimensions of the food system (16, 18, 44–47, 49, 50). Inclusion of the environmental impacts of the food system is a priority in the future development of the DELTA Model.

It should also be noted that the FAO FBS do not provide complete coverage of global food production. A total of 19 countries, mostly developing nations or low-volume food producers, are not currently included in the FBS. This likely results in a small underestimation bias for nutrient delivery, as the populations of these countries (totaling 182 million people) are included in the model, but not their food production.

Moreover, the FBS only give information on food supply and not consumption. We have addressed this issue by subdividing the FBS food items into more specific food types using detailed supply data for these. Finally, the FBS do not capture subnational food availability. We have not attempted to address regional nutrient availability, instead taking a global average approach, which masks the inequitable distribution of food in many parts of the world. Considering these limitations of the data, the model predictions are an estimate of global nutrient availability to the average global citizen. Despite these limitations, the FBS were chosen for use in the model as an international standardized data set, with annual updates to allow for continued relevance of the DELTA Model. It is hoped that the omitted countries and a greater resolution will be included in future FBS, allowing future versions of the model to provide increasing levels of accuracy.

There also exist limitations to the resolution of the model. Currently, food items are grouped into 15 food groups for ease of use by a broad audience. This means that any increase in the total production of a food group causes a proportional increase in the production of each food item within it, following the distribution in the baseline data set. This increase is carried through all model calculations: waste, other uses, and the available nutrients. Future versions of the model will allow for user input at a higher resolution.

The DELTA Model differs from much of the sustainable nutrition literature by not considering individual diets. The inputs and outputs of the model are for the global food system, considering the ways in which the world can feed the world. Much research exists on the environmental, economic, and health sustainability of food from an individual dietary perspective (15, 22, 25, 44, 47, 50, 53, 54, 60–62). Individuals, particularly in developed regions, have broad choices in achieving nutrient adequate diets. The DELTA Model demonstrates that achieving nutrient adequacy at a global scale has fewer possible solutions. Both the individual and the global perspectives, and thus both modeling approaches, are necessary when considering the aptitude of the food system. It is at the individual level that we choose foods to meet our own requirements, but on a global scale that we develop a food system that can provide nutrition to all.

An advantage of the DELTA Model over other similar models is its consideration of amino acids in addition to protein, as well as the digestibility of these nutrients. Foods cannot be assumed to deliver equal nutritional value purely because they have similar nutritional content (29). Differences in the bioavailability of nutrients must be considered when assessing nutrient adequacy of the diet, but this is highly dependent on data specific to each nutrient from each food source and dietary pattern. The DELTA Model utilizes digestibility data for protein and the IAA, but insufficient data exists for the bioavailability of other nutrients. Instead, the model utilizes iron and zinc target values for vegetarian and vegan diets when the scenario considered is predominantly or entirely plant based, respectively. This is an approximation and will not be appropriate in all cases: for example, zinc bioavailability is negatively impacted by phytate, an antinutritional compound found in plants (51). Therefore, zinc bioavailability will vary on an individual basis depending on the amount of phytate in the diet. Our assumption of a single, greater target daily intake for zinc in vegetarians and vegans appears to be the best manner of modeling variation in absorption at a global level given the dearth of digestibility data for individual foods.

There is increasing awareness of the need for the global food system to change in order to sustainably nourish the entire global population. Much of the narrative centers around significant shifts towards predominantly or entirely plant-based individual diets to achieve health and environmental goals (2, 5, 53). The DELTA Model demonstrates the numerous nutrient gaps that would emerge if food production followed these dietary trends. The results indicate the need for a balanced approach: both animal and plant foods provide essential nutrients, and our current food production system supplies almost all the nutrients needed by the global population. Emphasis should be placed on the system remaining plant based and animal optimized, and on improving both of these aspects. It is possible that moderate refinements to this system and more equitable distribution would achieve nutritional sustainability without the need for radical changes to unfamiliar production and dietary practices.

Many models have been created for different aspects of the food system and have begun to explore ideas for how it might be transformed. The DELTA Model provides an important contribution towards understanding the current problems of the food system and the implications of proposed changes on global nutrient availability. Nutrition must be a priority in the design of future food systems and the DELTA Model facilitates this consideration.

## Supplementary Material

nxab199_Supplemental_Files

## Notes

The authors reported no funding received for this study.

Author disclosures: The authors report no conflicts of interest.

Supplemental Figure 1 and Supplemental material (5 documents), including detailed descriptions of the methods, is available from the “Supplementary data” link in the online posting of the article and from the same link in the online table of contents at https://academic.oup.com/jn.

Abbreviations used: DRV, dietary reference value; EFSA, European Food Safety Authority; FBS, food balance sheets; IAA, indispensable amino acids; UN, United Nations.
