# Supplementary material for: Use of the DELTA Model to Understand the Food System and Global Nutrition
Source: J Nutr. 2021 Jun 30;151(10):3253–61. doi: 10.1093/jn/nxab199 (PMC8485910; doi:10.1093/jn/nxab199)
Supplement: nxab199_Supplemental_Files [file nxab199_supplemental_files.zip › SupplementaryFigure1.pdf]

Use of the DELTA Model to understand the food system and global nutrition
Smith et al.
Online Supplementary Material

Supplementary figure 1

A map of the global food system illustrating the complexity and interconnectedness of all its aspects. Consideration of the entire system is necessary in the design of sustainable future food systems. Reproduced with permission from ShiftN (https://shiftn.com/).

Global Food System Map

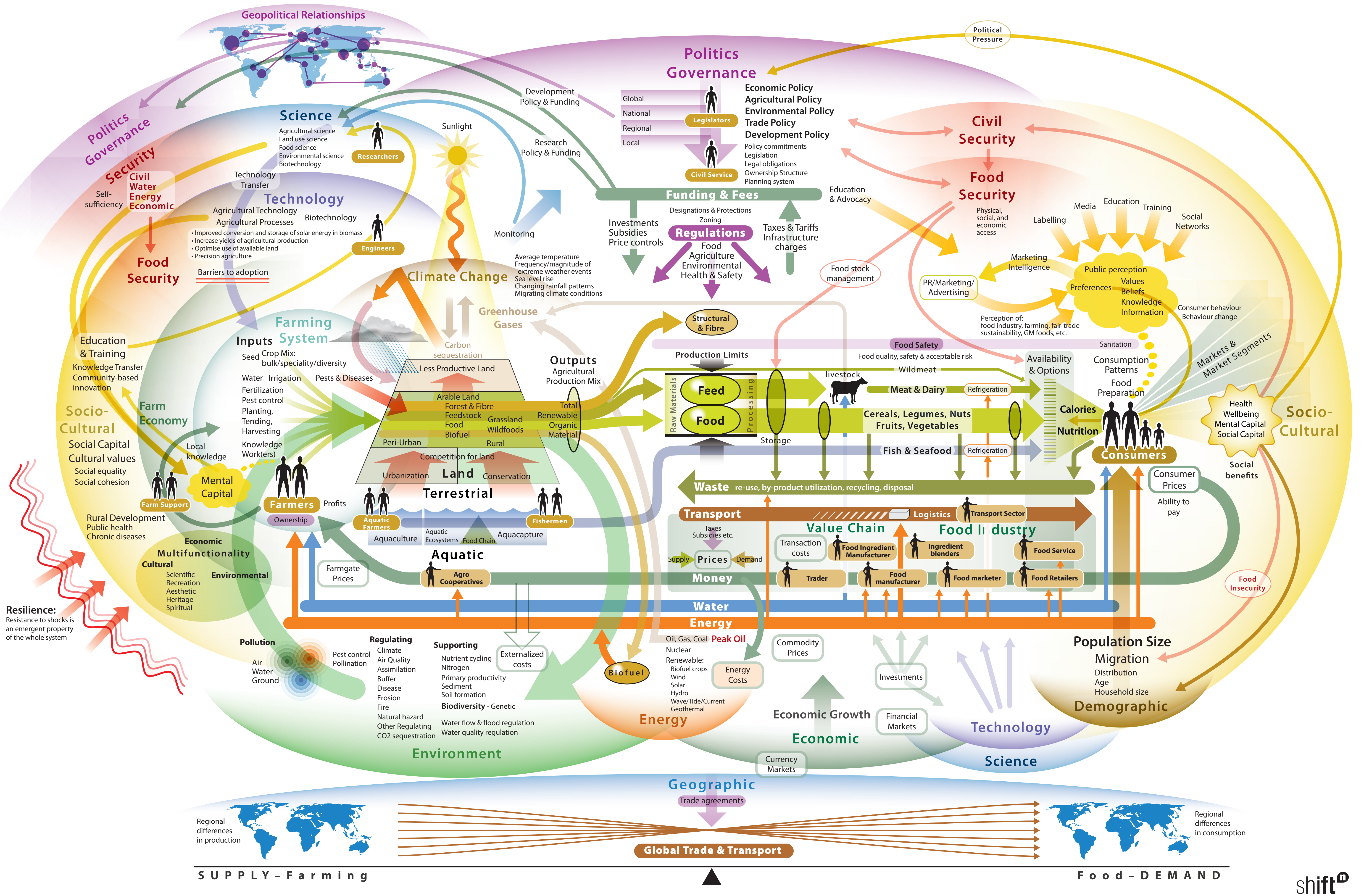

copyright® 2009 shiftn cvba

shiftn
clarity in complexity
