# Supplementary material for: Use of the DELTA Model to Understand the Food System and Global Nutrition
Source: J Nutr. 2021 Jun 30;151(10):3253–61. doi: 10.1093/jn/nxab199 (PMC8485910; doi:10.1093/jn/nxab199)
Supplement: nxab199_Supplemental_Files [file nxab199_supplemental_files.zip › SupplementaryMaterial1.pdf]

Population and Nutrient Targets

Population

Population data was taken from United Nations (UN) data and projections of world population by gender and age (in one-year age increments) from 2010 to 2100 (1). For use in the DELTA Model, this data was assembled into regional groups (Africa, America, Asia, Europe, Oceania), filtered to isolate the data for specific target years (2010, 2015, 2018, 2020, 2025, 2030, 2040, 2050), and further grouped into 21 age bands (0-11 months and then five-year bands thereafter, with the final band being 96+ years).

Bodyweight

Weight for age data from the European Food Safety Authority (EFSA) was used to generate a piecewise linear interpolation of weight for all ages from 0 – 100 years for both males and females (2).

Protein and amino acid requirements

Indispensable amino acid (IAA) and total protein requirements were determined by combining the age- and weight-based protein intake values.

Target daily protein intake per kg body mass was taken from the EFSA nutrition guidelines (2), which include an average requirement (AR) and a population reference intake (PRI) value. In the DELTA Model, the AR was used to generate the lower limit and the PRI the target level. The EFSA guidelines were chosen due to their international relevance and broad coverage of nutrients.

When converting this to IAA requirements, the relevant age-based overall IAA profile – a combination of the maintenance and tissue IAA patterns – was used for the lower limit (3). To calculate the IAA requirements for the target protein intake, the maintenance IAA pattern was applied to the additional protein.

The daily protein and IAA intake requirements were found by combining the age-based requirements (g kg^{-1} day^{-1}) with the bodyweight for age estimates.

Energy Requirements

Energy requirements in the EFSA dataset were specified based on a combination of gender, age and physical activity level (PAL) (2). Where only a single value for a gender-age combination was given, the same value was used for the lower, target and upper limit calculations. Where three PAL levels were specified, these were aligned with the lower, target and upper levels. Where four levels were given, the lowest was used for the lower limit, the second for the target, and the highest for the upper limit.

Energy-based Nutrient Requirements

Nutrient intake levels for some nutrients (e.g., Total carbohydrates, total fat, Niacin) were specified as a proportion of total energy intake. These values were given either as a fixed value or as a range (lower and upper values). In converting these to absolute requirements, the following approach was taken:

- The lower limit was set at the lower of the nutrient requirement values applied to the lower level for energy intake

- The upper limit was set at the upper of the nutrient requirement values applied to the upper level for energy intake
- The target level was set at the lower of the nutrient requirement values applied to the upper level for energy intake. Thus, an individual with energy intake at the upper end of the energy intake guidelines will meet the minimum proportion of the energy target for the nutrient.

General Nutrients

For nutrients with requirements given in absolute quantities, the following approach was applied. Where there was only a single value given – typically an adequate intake (AI) or AR value – this was used for both the lower and target levels within DELTA. Where there was both an AR and a PRI, the AR was used to set the lower level and the PRI to set the target. Upper levels were only set where there was a specified upper limit (UL).

Global Nutrient Intake Targets

Nutrient requirements were calculated for all ages from birth to 100+ years and then averaged for the five-year age bands. This gave a set of gender and age based nutrient intake targets. These targets were then combined with global demographic projections to derive a population weighted average requirement for each nutrient. This was performed for each year included in the DELTA Model.

Bioavailability

To address differences in the digestion and absorption of nutrients between different food sources and under different diets, two approaches have been applied in the DELTA Model.

1. Food item specific bioavailability coefficients
2. Adjustment to the nutrient intake targets

Food Item Specific Coefficients

The application of this approach in DELTA 1.2 was limited to the absorption of seven indispensable amino acids and protein, where there exists a body of scientific literature on protein digestibility coefficients. The coefficients were derived from data used for the digestible indispensable amino acid score (DIAAS) method for protein quality assessment, and include weight- and age- based targets for the amount of each amino acid required (3). A number between zero and one that reflects the fraction of a specific amino acid or protein that is typically absorbed from a food item was assigned to each food item for each of these nutrients.

The available bioavailability data in the literature captures more than 80% of global protein supply in the DELTA Model. To address the remaining gaps, the following approximation method, based on the levels of the food composition model (see Composition Model in the Supplementary Material) was used:

> Case 1 – There was no bioavailability coefficient available for a specific food type, but coefficients existed for other food types assigned to the same food item.

In this case, the bioavailability coefficient for the food type was set to the average of the available bioavailability coefficients for other food types assigned to the same food item, weighted by the relative contribution of these food types to the food item.

> Case 2 – There were no bioavailability coefficients available for any of the food types assigned to a food item, but coefficients existed for other food items assigned to the same food group.

In this case, the bioavailability coefficient for the food item was set to the average of the available bioavailability coefficients for other food items assigned to the same food group, weighted by the relative contribution of these food items to the food group.

> Case 3 – There were no bioavailability coefficients available for any of the food types or food items assigned to a food group.

In this case, a weighted average bioavailability coefficient based on the bioavailability of all other food groups was assigned.

Nutrient Intake Target Adjustment

The application of this approach in DELTA 1.2 was limited to iron and zinc. This approach increases the target levels for these nutrients based on the range of food items present in the global food supply to reflect the differences in absorption from plant- and animal-based foods. This approach was taken rather than an absorption coefficient since the EFSA RDIs for these nutrients are set based on intake, rather than absolute requirement. Thus, the nutrient target also had to reflect intake.

Average iron bioavailability under omnivorous, vegetarian and vegan diets was taken from the World Health Organisation (4). Taking the mid-point of the non-vegetarian range as a reference point allows calculation of a modifier to be applied to the nutrient targets under vegetarian (meat free) and vegan (animal product free) scenarios.

Diet Type	Fe bioavailability	RDI modifier
Non-vegetarian	15% (range 12-18%)	x1
Vegetarian	10%	x1.5 (=15/10)
Vegan	5%	x3 (=15/5)

Absorption of zinc is impacted by the level of phytate in the diet. In the EFSA targets, zinc intakes are specified against a range of phytate intakes – in the case of adults at 300, 600, 900 and 1200 mg day^{-1} phytate intake. Adjustment of the RDI target is based on a mixed diet with an assumed 600 mg day^{-1} of phytate and a vegetarian or vegan diet with 1200 mg day^{-1}. The combined outcome of this is a modifier of x1.37 for the zinc targets under these diets.

Level of Phytate Intake (mg day^{-1})	AR		PRI		Alignment with "Diets"
	Male	Female	Male	Female	
300	7.50	6.20	9.40	7.50	
600	9.30	7.60	11.70	9.30	Mixed Diet
900	11.00	8.90	14.00	11.00	
1200	12.70	10.20	16.30	12.70	Vegetarian and Vegan Diets

List of nutrients included in the DELTA Model

The following nutrients are currently included in the DELTA Model. The reason for the inclusion of each is a combination of their importance to human nutrition, their inclusion in the food composition data from USDA (5), and the availability of their target intakes or bodily requirements (2-4).

Energy
Protein

Dietary fat
Carbohydrate
Dietary fibre
Calcium
Iron
Magnesium
Phosphorus
Potassium
Selenium
Zinc
Copper
Thiamin
Riboflavin
Folate
Vitamin C
Pantothenic acid
Vitamin B-6
Vitamin B-12
Vitamin A
Vitamin E
Cystine
Histidine
Leucine
Lysine
Methionine
Threonine
Tryptophan

References

1. United Nations Department of Economic and Social Affairs Population Division. World Population Prospects 2019; 2019.
2. European Food Safety Authority. Dietary Reference Values for nutrients: Summary report. EFSA Supporting Publications. 2017;14:e15121E.
3. FAO. Dietary protein quality evaluation in human nutrition: United Nations Food and Agriculture Organisation; 2013.
4. World Health Organisation. Vitamin and mineral requirements in human nutrition. Second Edition ed. Bangkok, Thailand: WHO; 2004.
5. USDA. FoodData Central. 2020 [cited 21 August 2020]; Available from: https://fdc.nal.usda.gov/download-datasets.html
