# Supplementary material for: Use of the DELTA Model to Understand the Food System and Global Nutrition
Source: J Nutr. 2021 Jun 30;151(10):3253–61. doi: 10.1093/jn/nxab199 (PMC8485910; doi:10.1093/jn/nxab199)
Supplement: nxab199_Supplemental_Files [file nxab199_supplemental_files.zip › SupplementaryMaterial2.pdf]

Composition Model

This section explains the key components of the composition model.

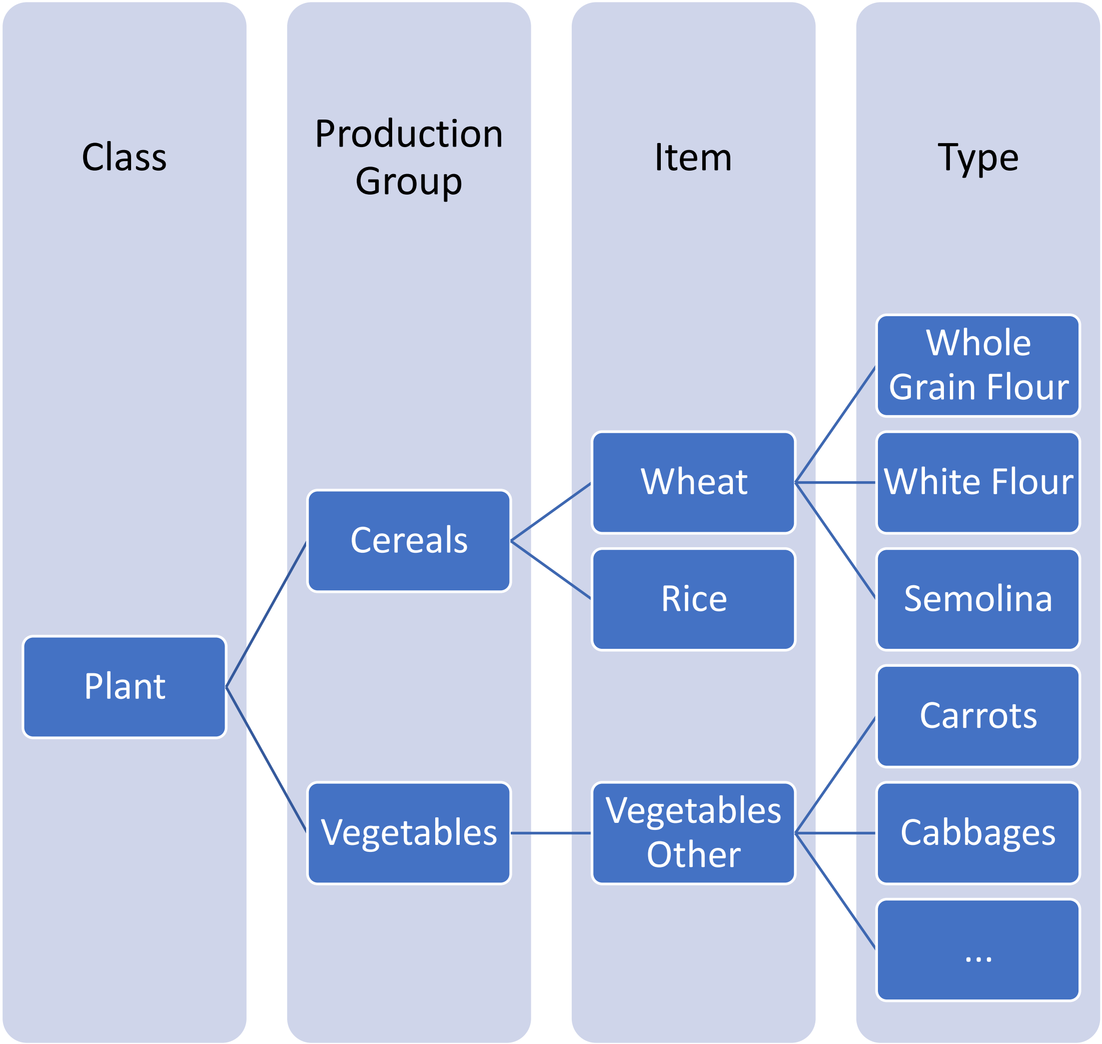

Figure S1. Schematic illustrating the hierarchy of classification in the DELTA Model using examples from food production groups Cereals and Vegetables.

Food Items

The primary unit of calculation within the model is a Food Item as defined in the FAO Food Balance Sheets (1).

Individual food items are categorised as being primary, secondary or tertiary commodities based on their position in the commodity processing chain. This is illustrated below in the case of the primary commodity sugar cane, which is processed into secondary commodities sugar and molasses, with both potentially processed into tertiary commodities alcoholic beverages and non-food alcohol.

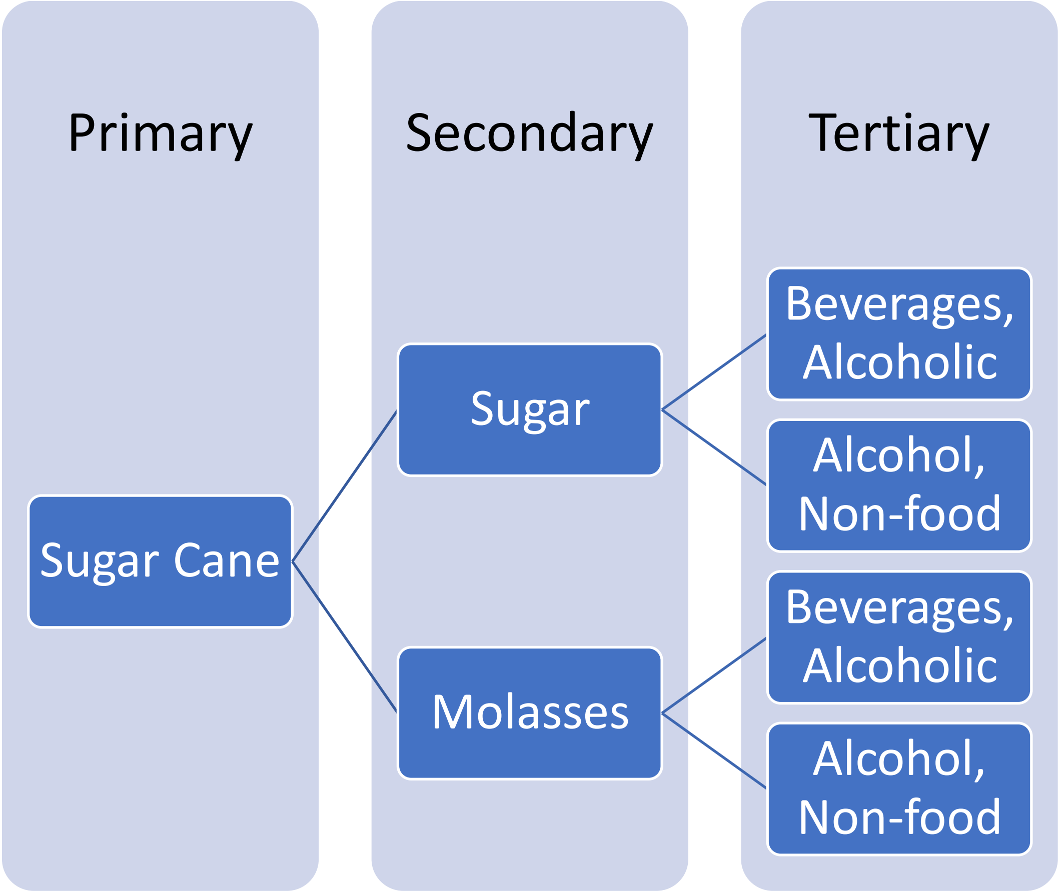

Figure S2. Schematic showing the examples of the primary, secondary and tertiary food items associated with sugar cane.

An additional group of items are primary by-products. These are items that are produced alongside and in fixed proportion with another primary commodity but not through the processing of that commodity. Examples include animal fats and offal. The model uses fixed ratios to estimate these based on the total production volume of the related primary item.

Food Production Groups

For the purpose of enabling the user to specify a food production system without the need to individually specify production levels for all 98 primary commodities, these are aggregated together into 15 food production groups – six related to animal production systems, and nine to plant systems.

User inputs on the production of each of these groups acts to scale all primary commodities within the group by the same ratio compared with the reference data set, e.g. if cereal production is increased by 50% from the base scenario, this is applied to each of barley, wheat, rice, etc.

The production of secondary and tertiary commodities is determined by the amounts of the parent commodities available for processing. The method by which this is determined is related in the supplementary material on the calculation sequence.

The model also includes a second set of food groups that correspond to the groups within the Food Balance Sheets. Whilst the food production groups relate to the nature of the primary commodity, this second arrangement relates to the nature of the food item and is used in displays of the aggregate contribution of food types to nutrition. An example is soyabean oil: from a production

perspective, this fits in the “oilcrops” group, but from a food perspective this is in the “vegetable oils” group, alongside other oils including rice bran and maize germ oils derived from cereals.

Food Types

To determine the nutrients supplied by food items, these items are subdivided into more specific food types (Figure 1). These types represent either different food materials that have, for convenience, been grouped together into a single food item, or different ways in which a basic commodity is used as food where this has an impact on nutrient delivery.

In some cases there exists a broad range of food types within a food item (e.g. the Vegetables Other food item includes: Cucumbers and gherkins, Cabbage, Eggplant, Carrots and parsnips, Turnips, Chillies and Peppers, Garlic, and several others), whilst in other cases there is only a single food type within a food item (e.g. Dates).

An example of where types have been used to represent differing uses of a food item (with different nutrient outcomes) is wheat. It is assumed that 20% of wheat is converted to whole grain flour, 60% to white flour, and 20% to semolina with an additional by-product of further white flour.

Where there are multiple food types attached to an individual item, each type has:

- a *weighting* that represents its relative production compared to the other types
- a *yield* that represents the amount of food material derived from a single unit of its respective base food commodity
- an *inedible portion* reflecting the fraction of the food type typically discarded in home.

The overall composition, yield, and inedible portion of the food item is a weighted average of the values for the component types. The major data sources for these values are public databases from the FAO and USDA and scientific publications (2-6); exact sources for each food item and type are displayed in the Composition Model page of the DELTA Model.

Two unusual cases are tea and coffee, where the yield of the food types is much greater than 1 as the nutritional composition refers to the beverage prepared with water rather than the base commodity. In the example of coffee, 1 kg of coffee beans results in approximately 50 kg of black coffee in the model.

Food Compositions

Each of the food types is further represented by one or more food compositions selected from the USDA food composition data base (5). It is this data that is used to calculate the total nutrient supplied by a food type, and subsequently a food item.

All the above information, including the resulting food compositions, yields and inedible portions, for each food item is visible within the DELTA model by selecting the Composition Model tab under the Helpful item on the top menu.

References

1. FAO. FAO Food Balance Sheets. 2020 [cited 21 August 2020]; Available from: http://www.fao.org/faostat/en/#data/FBSH
2. FAO. Global food losses and food waste - Extent, causes and prevention. Rome: United Nations Food and Agriculture Organisation,; 2011.
3. Food and Agriculture Organization. Yield and nutritional value of the commercially more important fish species. Rome; 1989.

4. United States Department of Agriculture Economic Research Service. Agricultural Handbook Number 697, Weights, Measures, and Conversion Factors for Agricultural Commodities and Their Products. Washington, DC: United States Department of Agriculture; 1992.
5. USDA. FoodData Central. 2020 [cited 21 August 2020]; Available from: https://fdc.nal.usda.gov/download-datasets.html
6. Smith MR, Micha R, Golden CD, Mozaffarian D, Myers SS. Global Expanded Nutrient Supply (GENuS) Model: A New Method for Estimating the Global Dietary Supply of Nutrients. PLoS ONE. 2016;11:e0146976.
