# Supplementary material for: Use of the DELTA Model to Understand the Food System and Global Nutrition
Source: J Nutr. 2021 Jun 30;151(10):3253–61. doi: 10.1093/jn/nxab199 (PMC8485910; doi:10.1093/jn/nxab199)
Supplement: nxab199_Supplemental_Files [file nxab199_supplemental_files.zip › SupplementaryMaterial3.pdf]

Use of the DELTA Model to understand the food system and global nutrition
Smith et al.
Online Supplementary Material

Calculation Methodology

The main source data for the model was the FAO food balance sheets (1). These contain information on the supply of food commodities at an individual country level, including production, imports, exports, domestic supply quantity, and draw on stocks. The food balance sheets also contain information on the uses of food commodities, including feed, food, processing, seed, losses and other uses. As the DELTA Model considers global food supply, the import, export and country level data were unnecessary, so these were aggregated. The aggregated data set from the food balance sheets will be referred to in this document as the reference dataset.

The reference data set described the 2018 global food system, as derived from a weighted linear interpolation of FAO food system data for every year from 1998 to 2017. The interpolation weighted the data from each year such that more recent years had the greatest weighting, and thus contributed the most to the 2018 data set (i.e. a weighting of 20 was given to the 2017 data, 19 to the 2016 data, 18 to the 2015 data, and so on).

Primary, secondary and tertiary commodities

Commodities within the food balance sheets can be divided into primary, secondary and tertiary commodities based on how far along the processing chain they are produced. For example, sugar cane is a primary commodity that is processed to make sugar, a secondary commodity. Sugar can be further processed to manufacture alcohol, a tertiary commodity.

The DELTA Model uses a push approach to estimate downstream commodity production. The user specifies the overall production quantity for groups of primary commodities. The amounts of secondary and tertiary commodities produced from this primary commodity are calculated from the quantities assigned to processing in the reference dataset.

For food item j that is made exclusively by processing another food item i:

$$Supply_j = Process_i \times Yield_{i,j}$$

Where $Supply_j$ (tonnes) is the total supply of food item j, $Process_i$ (tonnes) is the amount of food item i that is directed to processing and $Yield_{i,j}$ is a ratio for the amount of j produced per unit i processed. The yield coefficients for each food item are based on values derived from the food balance sheets.

If there are multiple processing options for producing j (e.g., sugar can be produced from both sugar cane and sugar beet), then the above equation is extended. For all food items $i = 1, \ldots, I$ that are processed to j:

$$Supply_j = \sum_{i=1}^{I} \left(Process_i \times Yield_{i,j} \right)$$

Co-products

In addition to processed commodities there are some entries in the food balance sheet that are scaled based on the production of other primary commodities without being a direct product of their processing. The main examples are animal fats, which occur in proportion to the production (or global supply quantity) of meat.

For a co-product j that results from production of primary commodity i:

$$Supply_j = Supply_i \times Coproduct_{i,j}$$

Here, $Coproduct_{i,j}$ is the number of tonnes of j produced per tonne of i produced, determined from the food balance sheets. This value is defined as:

$$Coproduct_{i,j} = \frac{{Supply_j}^{ref}}{{Supply_i}^{ref}}$$

where the superscript ref refers to values in the reference dataset.

Food Balance Sheet Calculations

Users specify food production in their scenarios by setting the global supply of the primary food commodities in 15 food production groups. For some user-defined scenario a, the total supply of an individual primary commodity i in food group g is:

$${Supply_i}^a = \frac{{Supply_g}^a}{{Supply_g}^{ref}} \times {Supply_i}^{ref}$$

A certain proportion of some food items are used as seed. The seed requirements are found in a similar manner:

$${Seed_i}^a = \frac{{Supply_g}^a}{{Supply_g}^{ref}} \times {Seed_i}^{ref}$$

where ${Seed_i}^a$ (tonnes) is the amount of food item i that is assigned to seed.

Feed allocation

After the production and seed use of primary commodities is established, the next stage is to calculate the change in the feed demands on the food system based on changes in primary production of animal-sourced foods.

Plant-sourced production that has no human food application (e.g., pasture, forage crops, hay) does not appear in the food balance sheets and is not captured by the DELTA Model. However, animals also consume a significant amount of potentially human edible material. The food balance sheets capture these uses in the Feed element. Note that some food items in the food balance sheets are processed to animal feed products not captured by the food balance sheets. These include the various oil seed cakes e.g., soyabean cake. These are not currently accounted for in the model due to the lack of data in the food balance sheets.

The DELTA Model uses data from the FAO on the global feed consumption of the most common domesticated animals to create mathematical linkages that enable estimation of the feed required to support different levels of animal-based production (2). The feed requirements are *pulled* through the model from the user set animal production values. For example, if the production of ruminant meat is doubled compared to the reference dataset, then the DELTA Model doubles the amount of grain allocated to ruminant feed compared to the reference dataset, reducing the amount available for all other uses.

For food item i that is used as feed to produce item j:

$${Feed_{i,j}}^a = {Supply_j}^a \times ReqFeed_{i,j}$$

where $Feed_{i,j}{}^{a}$ (tonnes) is the amount of food item i that is used as feed in the production of item j, and $ReqFeed_{i,j}$ is the number of tonnes of i required to produce one tonne of item j. In many cases feed supports production of more than one animal-sourced product. Thus, for animal-sourced products $j = 1, \dots, J$:

$$Feed_{i,j}{}^{a} = \sum_{j=1}^{J} \left(Supply_{j}{}^{a} \times ReqFeed_{i,j}\right)$$

To establish the feed requirement coefficients, the work of Mottet, de Haan (2) was used to determine the portion of each feed type consumed by the various animal production systems. This gives the set of feed use ratios shown in Table S1.

Table S1. Feed allocation across the DELTA model animal production systems

	**Feed Groups used to produce Food Group**						
**Food Group**	**Forage**	**Cereals**	**Oilseeds**	**Other Crops**	**By-products**	**Crop Residues**	**Animal Products**
Dairy	33%	11%	8%	0%	29%	32%	0%
Ruminant Meat	67%	23%	16%	0%	57%	64%	0%
Eggs	0%	14%	14%	7%	1%	0%	19%
Poultry	0%	31%	32%	15%	3%	0%	43%
Other Meat	0%	21%	30%	78%	10%	3%	38%

The following additional assumptions were made:

- Global milk production represents 33% of the feed use by ruminants
- Global egg production represents 31% of the feed use by poultry[1]
- Use of animal products in feed is restricted to Eggs, Poultry and Other Meat with 38% going to Other Meat[2]
- Milk consumed as animal feed goes entirely into dairy
- Seafood consumed as animal feed goes entirely into aquaculture

The feed use ratios are immediately applied to all items in the food balance sheet based on the Feed Group they have been assigned to. For example, if increased animal production in the modelled scenario results in an increased feed demand for cereals, then the feed allocations for all the cereal crops (wheat, barley, rice, etc…) will be increased by the same proportion.

Primary commodity calculations

The calculation sequence continues by solving for the primary commodity balances.

Supply chain losses

Once the feed allocations have been made the remaining primary commodity supply is allocated amongst the other outcomes. The first of these is supply chain losses. Supply chain losses are calculated as a proportion of the remaining supply after the allocation of feed and seed. These are adjusted based on the loss modifier (R_Loss^{a}, taking values between 0 and 1.5) set by the user. The default loss value ($R\_Loss^{a} = 1$) is that of the reference dataset.

[1] Based on the ratio of total solids between eggs and poultry meat – assuming 1 g of egg solids requires the same feed input as 1 g of meat solids.
[2] Retains the same ratio as the rest of the feed.

The supply chain losses of a food item are calculated as:

$$Loss_i^{\,a} = R\_Loss^a \times \frac{Supply_i^{\,a} - Feed_i^{\,a} - Seed_i^{\,a}}{Supply_i^{\,ref} - Feed_i^{\,ref} - Seed_i^{\,ref}} \times Loss_i^{\,ref}$$

where R_Loss^a (tonnes) is the amount of food item i that is lost along the supply chain in scenario a and $Loss_i^{\,ref}$ (tonnes) is the same variable for the reference dataset. This yields $Unalloc_i^{\,a}$ (tonnes), the quantity of food item i still available for use:

$$Unalloc_i^{\,a} = Supply_i^{\,a} - Feed_i^{\,a} - Seed_i^{\,a} - Loss_i^{\,a}$$

Primary commodity non-food, food and processing uses

The next step is evaluation of the amount of the available food item that is used for non-food purposes (e.g., biofuels, soap). The user selects how this is allocated from three options:

1. Non-food use remains ***constant***. This retains the mass allocation to non-food use from the reference dataset
2. Non-food use ***scales with population,*** i.e., if the population increases by 10% then so does each non-food use
3. Non-food use is allocated as a ***fraction of the available food item,*** i.e., if the available food item quantity increases by 10% then so does each non-food use.

Constant and Population-based allocation

Options 1 and 2 above are mathematically similar. $Other_i^{\,a}$ (tonnes), the amount of food item i allocated to other uses in scenario a, is defined as:

$$Other_i^{\,a} = R\_other^a \times Other_i^{\,ref}$$

R_other^a is the other use ratio which takes the value 1 for option 1 above, and for option 2 is calculated from the ratio of the scenario population (N^a) to the reference dataset population (N^{ref}):

$$R\_other^a = \frac{N^a}{N^{ref}}$$

The amount of the food item remaining is updated by removing the quantity taken for other uses:

$$Unalloc_i^{\,a} \leftarrow Unalloc_i^{\,a} - Other_i^{\,a}$$

For both options 1 and 2 above, the amounts of food item i that go directly to food ($Food_i^{\,a}$ (tonnes)) or to further processing are calculated as:

$$Food_i^{\,a} = \frac{Unalloc_i^{\,a}}{Unalloc_i^{\,ref}} \times Food_i^{\,ref}$$

$$Process_i^{\,a} = \frac{Unalloc_i^{\,a}}{Unalloc_i^{\,ref}} \times Process_i^{\,ref}$$

Availability-based allocation

For non-food option 3 above, the quantity directed to other uses is calculated simultaneously with the food and processing values:

$$Other_i^{\,a} = \frac{Unalloc_i^{\,a}}{Unalloc_i^{\,ref}} \times Other_i^{\,ref}$$

$$Food_i^{\ a} = \frac{Unalloc_i^{\ a}}{Unalloc_i^{\ ref}} \times Food_i^{\ ref}$$

$$Process_i^{\ a} = \frac{Unalloc_i^{\ a}}{Unalloc_i^{\ ref}} \times Process_i^{\ ref}$$

Co-product balances

The equation determining the value for the co-products of the primary commodities has already been presented (see Co-products above). Any feed use of these co-products has already been assigned as part of the feed calculations, thus only supply chain losses, other uses, food and processing quantities need to be calculated. The process for doing so is identical to that used for primary commodities in the previous section.

Secondary commodities

The calculation process continues by stepping along the production chain to solve the item balances at each supply chain stage. The supply of secondary commodities is calculated based on the processing quantities of the relevant primary commodities using the equations described earlier and the allocations repeated as for co-products. This continues for as many stages along the supply chain as are required to solve for all the items in the balance sheet (i.e., for secondary commodities, tertiary commodities, etc.). The DELTA Model currently only includes tertiary commodities but adding additional steps in the supply chain would be straightforward.

At this point a scenario balance sheet covering food items from farm to retail has been established.

In-home waste

The final element of the scenario balance is the allocation of food item quantities between material wasted in home or used as food. All food items have been allocated to a Waste Group (*w*) (3). The domestic waste ratios differ between geographic region and food group, as shown in Table S2. The user sets a value for the in-home waste modifier, R_waste (between 0 and 1.5) as a multiple of the reference in-home waste rate (WR).

$$Waste_i^{\ a} = R\_waste^a \times \frac{\sum_r \left(N_r^{\ a} \times WR_{w,r}\right)}{N^a} \times Food_i^{\ a}$$

where $Waste_i^{\ a}$ (tonnes) is the amount of food item i that is wasted in-home in scenario a. $WR_{w,r}$ is the in-home waste rate of food items in waste group w in global region r. The quantity of food item i remaining in scenario a is then updated:

$$Food_i^{\ a} \leftarrow Food_i^{\ a} - Waste_i^{\ a}$$

Table S2. In-home losses as a fraction of food item mass by food Waste Group and global region (see (3))

Waste Group	Africa	Americas	Asia	Europe	Oceania[3]
Cereals	0.01	0.27	0.2	0.25	0.27
Fish and seafood	0.02	0.33	0.08	0.11	0.33
Fruits and vegetables	0.05	0.28	0.15	0.19	0.28
Meat	0.02	0.11	0.08	0.11	0.11

[3] Oceania values are a copy of the American values rather than the result of any independent analysis

Milk	0.001	0.15	0.05	0.07	0.15
Oilseeds and pulses	0.01	0.04	0.04	0.04	0.04
Roots and tubers	0.02	0.3	0.1	0.17	0.3

Nutrition Delivery Calculation

The calculations above result in an estimate for the quantity of each food item that reaches the point of consumer food preparation. The DELTA Model next calculates the nutrient delivery from this food.

Inedible Portions

Not all the biomass that reaches consumer food preparation can be eaten. Many foods at this stage still contain a significant portion of inedible material that is discarded as part of the final preparation process (e.g., skins of many fruits and vegetables, bones of meat and fish). The discarding of this material is not included in the in-home waste calculation above, so must be handled separately.

To adjust for inedible portions, there is a further reduction in food item quantity. The USDA has published data on the inedible portion of a wide range of food items (4).

The inedible fraction of food item i is given by $Inedible_i$. ${Eaten_i}^a$ (tonnes), the amount of food item i that is available for consumption in scenario a, is thus:

$${Eaten_i}^a = (1 - Inedible_i) \times {Food_i}^a$$

Nutrient Delivery

Having arrived at a value for the mass of each food item available for consumption on a global scale, the DELTA Model next determines the nutrient supply this represents. Two key factors in this are the nutrient content of the food, and the bioavailability of that nutrient.

Food composition data was sourced from the USDA food composition database (4). The annual global nutrient intake is thus:

$${Intake_n}^a = \sum_i {Eaten_i}^a \times X_{i,n} \times Bioavailability_{i,n}$$

Where ${Intake_n}^a$ is the total annual intake of nutrient n in scenario a, $X_{i,n}$ is the content of nutrient n in food item i, and $Bioavailability_{i,j}$ is the coefficient capturing the bioavailability of nutrient n in food item i. Measurement units from this point are dependent on the unit used to measure each nutrient. Note that this sum is over all food items modelled.

Global averaging

The final stage of the calculation is to divide the annual nutrient intake by the global population and the length of the year to get the average daily intake for the hypothetical global citizen:

$${Daily_n}^a = \frac{{Intake_n}^a}{365 \times N^a}$$

where ${Daily_n}^a$ is an individual's daily intake of nutrient n in scenario a. This can then be compared with the population weighted recommended daily intake (RDI) targets to determine the extent to which the global food system can meet the needs of the population in the given scenario. See

Supplementary Material on bioavailability for the derivation of these coefficients and on population for derivation of the RDI targets.

Table of Nomenclature

**Model value**	**Unit**	**Description**
Variables		***Food balance components***
$Supply_i$	tonnes	The total supply of a food item (or group) i. This is equal to the global sum of the Domestic supply quantity as defined in the Food Balance Sheets. Note, if given with subscript g, refers to the supply of food group g.
$Feed_{i,j}$	tonnes	The amount of food item i used as feed for animals in the production of item j
$Food_i$	tonnes	The amount of food item i available for use as human food
$Seed_i$	tonnes	The amount of food item i used as seed to enable future production
$Process_i$	tonnes	The amount of food item i that is processed to produce other food items also captured in the Food Balance Sheets
$Other_i$	tonnes	The amount of food item i that is used for non-food purposes (e.g., manufacture of biofuels, soaps, pet-food)
$Loss_i$	tonnes	The amount of food item i lost along the formal supply chain between the farm and retail
$Waste_i$	tonnes	The amount of food item i wasted in-home
		User set values
R_Loss	-	The user specified ratio of the loss rate in the scenario relative to the reference data set
R_waste	-	The user specified ratio of the level of in-home waste in the scenario relative to the reference data set
R_other	-	A ratio that results from the user selection of one of three options for how non-food uses are estimated
		Other variables
$ReqFeed_{i,j}$	tonnes	The amount of food item i required as feed to produce one tonne of food item j
$Unalloc_i$	tonnes	The amount of food item i that is unallocated at the point where the allocation between non-food uses, processing, and food occurs
$Eaten_i$	tonnes	The amount of food item i that is available for consumption after consideration of all food balance deductions, in-home waste, and inedible portions
$Intake_n$	Nutrient specific unit	Global population supply of a bioavailable nutrient n
$Daily_n$	Nutrient specific unit	Daily intake of a bioavailable nutrient n for the average global citizen
N_r	Persons	Population in region r
Constants		

$Yield_{i,j}$	-	The ratio between the amount of food item j that is produced as a result of the total processing allocation of food item i
$Coproduct_{i,j}$	-	The ratio between the amount of food item j that is produced as a co-product of production of food item i
$WR_{w,r}$	-	The fraction of food items in the waste group w that is wasted in-home in region r
$Inedible_i$	-	The fraction of food item i that is inedible and discarded before consumption
$X_{i,n}$	Nutrient specific unit	The amount of nutrient n contained within a standard amount of food item i.
$Bioavailability_{i,n}$	-	The fraction of nutrient n contained within food item i that is effectively available for use within the body
Superscripts		
ref	-	Denotes that the quantity is taken from the reference dataset
a	-	Denotes that the quantity refers to scenario a

References

1. FAO. FAO Food Balance Sheets. 2020 [cited 21 August 2020]; Available from: http://www.fao.org/faostat/en/#data/FBSH
2. Mottet A, de Haan C, Falcucci A, Tempio G, Opio C, Gerber P. Livestock: On our plates or eating at our table? A new analysis of the feed/food debate. Global Food Security. 2017 2017/09/01/;14:1-8.
3. FAO. Global food losses and food waste - Extent, causes and prevention. Rome: United Nations Food and Agriculture Organisation,; 2011.
4. USDA. FoodData Central. 2020 [cited 21 August 2020]; Available from: https://fdc.nal.usda.gov/download-datasets.html
