# Supplementary material for: Use of the DELTA Model to Understand the Food System and Global Nutrition
Source: J Nutr. 2021 Jun 30;151(10):3253–61. doi: 10.1093/jn/nxab199 (PMC8485910; doi:10.1093/jn/nxab199)
Supplement: nxab199_Supplemental_Files [file nxab199_supplemental_files.zip › SupplementaryMaterial4.pdf]

Use of the DELTA Model to understand the food system and global nutrition
Smith et al.
Online Supplementary Material

Initial values

The following document contains the DELTA Model inputs used for the scenarios described in the main text.

2018 baseline scenario

Year	2018
Reference RDI	Base
**Primary production (millions of tonnes)**	
Ruminant Meat	83.3
Poultry Meat	120.9
Other Meats	129.1
Eggs	86.2
Dairy	803.2
Fish/Seafood	212.2
Cereals	2908.8
Fruit	711.5
Nuts	16.7
Oilcrops	939.9
Pulses	84.8
Starchy Roots	855.7
Sugar	2297.9
Vegetables	1259.9
Other Plants	38.3
**Secondary modifiers**	
Supply chain waste	1x
In-home waste	1x
Include bioavailability	Yes
Scale of other uses	Population

2030 baseline

Identical to the 2018 baseline scenario, but with Year set to 2030, which increases the global population from 7.6 billion to 8.6 billion.

Food waste scenarios

Identical to the 2018 baseline scenario, but with supply chain waste and in-home waste varied, as displayed in the main text.

Scale-up 2030 scenario

Similar to the 2018 baseline scenario, but with Year set to 2030 and all primary production increased by 12%.

Year	2030
Reference RDI	Base
**Primary production (millions of tonnes)**	
Ruminant Meat	93.3
Poultry Meat	135.4
Other Meats	144.6
Eggs	96.5
Dairy	899.6
Fish/Seafood	237.7
Cereals	3257.9
Fruit	796.9
Nuts	18.7
Oilcrops	1052.7
Pulses	95
Starchy Roots	958.4
Sugar	2573.6
Vegetables	1411.1
Other Plants	42.9
**Secondary modifiers**	
Supply chain waste	1x
In-home waste	1x
Include bioavailability	Yes
Scale of other uses	Population

No meat 2030 scenario

Taking the 2018 baseline scenario as a starting point, all meat and seafood were set to zero, while the remaining primary production was increased by 20%. Year was set to 2030 and the reference RDI was set to vegetarian.

Year	2030
Reference RDI	Vegetarian
**Primary production (millions of tonnes)**	
Ruminant Meat	0
Poultry Meat	0
Other Meats	0
Eggs	103.4
Dairy	963.8
Fish/Seafood	0
Cereals	3490.6
Fruit	853.8
Nuts	20
Oilcrops	1127.8
Pulses	101.8
Starchy Roots	1026.8
Sugar	2757.5
Vegetables	1511.9
Other Plants	46
**Secondary modifiers**	
Supply chain waste	1x
In-home waste	1x
Include bioavailability	Yes
Scale of other uses	Population

No sugar 2030 scenario

Taking the 2018 baseline scenario as a starting point, sugar production was set to zero, and the biomass removed (2.3 billion tonnes) was allocated proportionally across the remaining plant groups. The primary production of animal food groups was increased by 12% to match the population increase. Year was set to 2030.

Year	2030
Reference RDI	Base
**Primary production (millions of tonnes)**	
Ruminant Meat	93.3
Poultry Meat	135.4
Other Meats	144.6
Eggs	96.5
Dairy	899.6
Fish/Seafood	237.7
Cereals	4356.3
Fruit	1065.6
Nuts	25
Oilcrops	1407.6
Pulses	127
Starchy Roots	1281.5
Sugar	0
Vegetables	1886.8
Other Plants	57.4
**Secondary modifiers**	
Supply chain waste	1x
In-home waste	1x
Include bioavailability	Yes
Scale of other uses	Population

Half waste 2050 scenario

Identical to the 2018 baseline scenario, but with both supply chain waste and in-home waste set to 0.5x. Year was set to 2050.

Bioavailability scenarios

Identical to the no meat 2030 scenario, but with Year set to 2050. Bioavailability was set either on or off, to compare the nutrient gaps.
